# The Tien Shan vole (*Microtus ilaeus*; Rodentia: Cricetidae) as a new species in the Late Pleistocene of Europe

**DOI:** 10.1002/ece3.8289

**Published:** 2021-11-04

**Authors:** Mateusz Baca, Danijela Popović, Anna Lemanik, Helen Fewlass, Sahra Talamo, Jan Zima, Bogdan Ridush, Vasil Popov, Adam Nadachowski

**Affiliations:** ^1^ Centre of New Technologies University of Warsaw Warszawa Poland; ^2^ Institute of Systematics and Evolution of Animals Polish Academy of Sciences Kraków Poland; ^3^ Department of Human Evolution Max Planck Institute for Evolutionary Anthropology Leipzig Germany; ^4^ Department of Chemistry G. Ciamician University of Bologna Bologna Italy; ^5^ Institute of Vertebrate Biology Academy of Sciences of Czech Republic Brno Czech Republic; ^6^ Department of Physical Geography, Geomorphology and Paleogeography Yuriy Fedkovych Chernivtsi National University Chernivtsi Ukraine; ^7^ Institute of Biodiversity and Ecosystem Research Bulgarian Academy of Sciences Sophia Bulgaria

**Keywords:** ancient DNA, grey voles, Late Pleistocene, mitochondrial DNA, Tien Shan vole

## Abstract

Grey voles (subgenus *Microtus*) represent a complex of at least seven closely related and partly cryptic species. The range of these species extends from the Atlantic to the Altai Mountains, but most of them occur east of the Black Sea. Using ancient DNA analyses of the Late Pleistocene specimens, we identified a new mtDNA lineage of grey voles in Europe. Phylogenetic analysis of mitochondrial DNA cytochrome *b* sequences from 23 voles from three caves, namely, Emine–Bair–Khosar (Crimea, Ukraine), Cave 16 (Bulgaria), and Bacho Kiro (Bulgaria), showed that 14 specimens form a previously unrecognized lineage, sister to the Tien Shan vole. The average sequence divergence of this lineage and the extant Tien Shan vole was 4.8%, which is similar to the divergence of grey vole forms, which are considered distinct species or being on the verge of speciation; *M*. *arvalis* and *M*. *obscurus* or *M*. *mystacinus* and *M*. *rossiaemeridionalis*. We estimated the time to the most recent common ancestor of the grey voles to be 0.66 Ma, which is over twice the recent estimates, while the divergence of the extant Tien Shan vole and the new lineage to be 0.29 Ma. Our discovery suggests that grey voles may have been more diversified in the past and that their ranges may have differed substantially from current ones. It also underlines the utility of ancient DNA to decipher the evolutionary history of voles.

## Graphical abstract

We sequenced mtDNA from multiple Late Pleistocene vole specimens from three caves near the Black Sea. We discovered a new divergent lineage of grey voles (subg. *Microtus*).

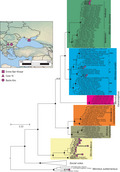

## 1 INTRODUCTION

Climatic and environmental changes in the Pleistocene are widely understood to have shaped the species evolutionary history and distribution of mammals worldwide (Hofreiter & Stewart, 2009; Stuart, 2015). The climatic and environmental processes that occurred during the Late Pleistocene and the onset of the Holocene had a profound impact on the extinction and formation of species that make up extant mammal faunas (Baca et al., 2017; Cooper et al., 2015; Lorenzen et al., 2011; Sommer, 2020). Small mammals are an integral component of every biome, and rodents, which are well‐known arvicolines (subfamily *Arvicolinae*), are the main component of nearly every Late Pleistocene fossil assemblage in the Northern Hemisphere. In Europe, aside from lemmings (*Dicrostonyx* and *Lemmus*), several vole species (from the genera *Arvicola*, *Lasiopodomys*, *Alexandromys*, and *Microtus*) are the predominant type of small mammal (Kowalski, 2001).

At present, meadow voles (genus *Microtus*) encompass nearly 60 species belonging to several subgenera spread throughout the entire Palaearctic and Nearctic (Jaarola et al., 2004; Pardiñas et al., 2017). Grey voles (subgenus *Microtus*) consist of at least seven closely related and partly cryptic species, namely, *M*. *arvalis* (Pallas, 1779), *M*. *obscurus* (Eversmann, 1841), *M*. *mystacinus* (de Filippi, 1865), *M*. *rossiaemeridionalis* Ognev, 1924 (= *M*. *levis* Miller, 1908; sensu Golenishchev et al., 2019), *M*. *transcaspicus* Satunin, 1905, *M*. *kermanensis* Roguin, 1988, and *M*. *ilaeus* Thomas, 1912; these species are often referred to as the ‘*M*. *arvalis* species group’ (Mahmoudi, Darvish, et al., 2017) and distributed in Europe and Western and Central Asia. The specific status of members of this group remains a matter of taxonomic debate. For example, some authors consider *M*. *arvalis* and *M*. *obscurus* as separate species (Tougard et al., 2013), whilst others consider them chromosomal races of the single species (Golenishchev et al., 2019; Sibiryakov et al., 2018). Similarly, the taxonomic status of the 54‐chromosome forms of grey voles, that is, *M*. *rossiaemeridionalis* and *M*. *mystacinus*, is ranked differently. Some authors treat these chromosomal forms as the single species *M*. *mystacinus* (e.g., Mahmoudi, Darvish, et al., 2017), whilst others reserve this species name only for the Iranian clade and classify European populations to the separate cryptic species *M*. *rossiaemeridionalis* (Golenishchev et al., 2019). In the Late Pleistocene of south‐eastern Europe, only two species of the *arvalis* group are recognized, namely, the common vole (*M*. *arvalis*), which has been detected in the Balkans and Pannonian Basin (e.g., Bogićević et al., 2017; Luzi et al., 2019; Mauch Lenardić, 2007; Popov, 2018) but is absent in the western and northern fringes of the Black Sea (Krokhmal & Rekovets, 2010; Petculescu & Ştiucă, 2008), and *M*. *obscurus*, which has been detected only in Crimea (Markova, 2011; Ridush et al., 2013).

The species determination as *M*. *arvalis* based on morphology has recently been confirmed by genetic studies of sub‐fossil specimens from different parts of Europe (Baca et al., 2020; Lemanik et al., 2020). Given the morphological and morphometric uniformity of molars of species from the *arvalis* group (Kochev, 1986; Markova et al., 2010), the timing of evolutionary events within the subgenus is based on molecular data. However, estimates of divergence times vary considerably depending on the calibration method used. The age of the most recent common ancestor of grey voles was estimated to be between 1.2 Ma, as determined using fossil calibration (Thanou et al., 2020), and 0.315 Ma, as determined using ancient DNA‐based calibration (Mahmoudi, Darvish, et al., 2017). Unfortunately, the remarkable variation in the estimates of species divergence prevents the accurate association of evolutionary events with biogeographic or climatic data and the reconstruction of the evolutionary history of this group.

The current study presents the results of ancient DNA analyses of *Microtus* s. str. from areas adjacent to the Black Sea. Amongst the sub‐fossil specimens from Bulgaria and Crimea described as *M*. *arvalis* or *M*. *obscurus*, we found specimens forming a divergent lineage of *M*. *ilaeus*.

## 2 MATERIALS AND METHODS

### 2.1 Samples

We investigated 47 specimens (Table S1) from various layers of three sites, namely, Emine–Bair–Khosar (Crimea, Ukraine), Bacho Kiro (Bulgaria), and Cave 16 (Temnata–Pochorna cave system, Bulgaria). We sampled isolated molars or mandibles with molars identified as *M*. *arvalis* or *Microtus* sp. on the basis of morphology. To increase the number and temporal range of radiocarbon‐dated specimens, we included two common vole mandibles, one from Obłazowa cave (WE) and another from Obłazowa 2 in Poland. Finally, we extracted DNA from ethanol‐preserved tissue fragments of four Tien Shan voles collected by JZ in the early 1990s in Kyrgyzstan and stored at the Institute of Vertebrate Biology, Czech Academy of Sciences, Brno, Czechia (Table S1).

### 2.2 Morphometric analyses

The morphological characteristics and general taxonomy of the fossil specimens, including those belonging to the newly identified mtDNA lineage (*n* = 14), were identified using linear measurements. The nomenclature and measurements of the first lower molar, total length (*L*), and length of the anteroconid complex (*A*) were conducted according to the methods proposed by van der Meulen (1973). La/Li indices were calculated to quantify the degree of asymmetry between triangles T4 and T5 according to the method prescribed by Nadachowski (1984a) and modified by Cuenca‐Bescós and Laplana (1995). This morphometric parameter is useful to distinguish vole species with an arvaloid morphology (e.g., Bogićević et al., 2012; Luzi & López‐García, 2019; Luzi et al., 2019; Nadachowski, 1984a, 1984b).

### 2.3 DNA extraction, enrichment, and sequencing

The samples were processed in a clean laboratory at the Laboratory of Paleogenetics and Conservation Genetics, Centre of New Technologies, University of Warsaw. The pre‐PCR laboratory has separate rooms designated for specimen preparation, DNA extraction, and library preparation. There is an increased air pressure to enforce unidirectional airflow toward the exterior of the laboratory, and the rooms were UV irradiated after each use. No modern tissues of any species have ever been processed in this laboratory. The post‐PCR laboratory is physically separated from the pre‐PCR one. Prior to DNA extraction, each tooth was rinsed with ultrapure water and crushed with a pipette tip. DNA was extracted using a protocol optimized for the retrieval of ultrashort DNA sequences (Dabney et al., 2013). Each batch of extractions was accompanied by blanks to monitor possible contaminations. A fraction of the extract was converted into double‐stranded, double‐indexed Illumina libraries following the protocol of Meyer and Kircher (2010) with minor modifications (Baca et al., 2019). After the fill‐in reaction, each library was amplified for 19 cycles in three replicates by using AmpliTaq Gold 360 DNA polymerase (Applied Biosystems). To enrich the mtDNA libraries, we performed two cycles of in‐solution hybridization following the protocol by Horn (2012).

The hybridization bait was composed of mtDNAs from *M*. *arvalis*, *M*. *agrestis* (Linnaeus, 1761), *Lasiopodomys gregalis* (Pallas, 1779), *Alexandromys oeconomus* (Pallas, 1776), and *Clethrionomys glareolus* (Schreber, 1780) to enrich the mtDNA of various *Microtus* species efficiently. The libraries were hybridized in pools of five. Each cycle of hybridization was conducted for 22–24 h, and the library pools were amplified in three replicates for 12–15 cycles after each round of hybridization by using Herculase II Fusion DNA Polymerases (Agilent). After hybridization, the libraries were quantified, pooled, and sequenced on the NextSeq550 platform (2 × 75 bp, MID output).

DNA from modern specimens was extracted in a laboratory that was physically isolated from the ancient DNA and post‐PCR laboratories by using a DNeasy blood & tissue kit (QIAGEN^®^) following the manufacturer's recommendations. A fraction of the DNA extracts was converted into sequencing libraries and subjected to in‐solution target enrichment by using the same protocols conducted to obtain libraries from the ancient DNA except that the new libraries were amplified for 12, instead of 19, cycles and only one round of hybridization was performed.

### 2.4 Sequencing read processing

Sequencing reads were demultiplexed using bcl2fastq (Illumina). Adaptors and low‐quality nucleotides were removed, and overlapping reads were merged using AdapterRemoval v.2 (Schubert et al., 2016). Filtered reads were mapped to the mtDNA sequences of various *Microtus* species using *bwa mem* (Li, 2013). Here, only reads longer than 30 bp and MapQ >30 were retained. Putative PCR duplicates were removed with the *samtools rmdup* command (Li et al., 2009). Comparison of mapping statistics with different mtDNA references enabled the preliminary species assignment. Variants were called using the *bcftools mpileup* and *call* commands, and alignments were visually inspected in Tablet (Milne et al., 2013). A list of regions with a coverage of <3 was generated using the *bedtools genomecov* command (Quinlan & Hall, 2010), and these regions were subsequently masked with N. Consensus was called using the *bcftools consensus* command. The mtDNA genome of extant *M*. *ilaeus* was *de novo* assembled using NOVOplasty (Dierckxsens et al., 2017).

### 2.5 Phylogenetic analyses

Phylogenetic reconstruction was conducted based on a sequence of the mtDNA cytochrome *b* (1143 bp). We used a dataset of 152 sequences that included all species of *Microtus* (s. str.), as well as species from the subgenera *Sumeriomys* (social voles) and *Terricola* (*M*. *subterraneus* (Selys‐Longchamps, 1836)). We used two sequences of field vole (*M*. *agrestis*) and another two sequences of European snow vole (*Chionomys nivalis* (Martins, 1842)) as out‐groups (Table S2). We accepted only sequences covering over 70% of the mtDNA cytochrome b. To include also available genetic information from *M*. *ilaeus igromovi*, a vicariant population of *M*. *ilaeus* classified as a subspecies (Golenishchev et al., 2019), additional analysis was performed using short cytochrome b fragment (341 bp).

The best‐fitting substitution model was revealed by jModelTest2 (Darriba et al., 2012) to be TrN+I+G. Bayesian phylogeny was reconstructed using MrBayes 3.2.7a (Ronquist et al., 2012). We conducted two independent runs with four coupled chains each for 5×10^6^ generations sampled every 500 generations. A maximum likelihood tree was reconstructed using IQtree (Nguyen et al., 2014) with 1000 ultrafast bootstrap replicates to assess branch support. In both analyses, the data were partitioned into three codon positions.

We used the Bayesian approach implemented in BEAST 1.10.4 (Suchard et al., 2018) to estimate divergence times within the *Microtus* subgenus. In this analysis, we used only sequences with known sampling times (*n* = 95; Table S2). To calibrate the molecular clock, we used 12 sequences obtained from directly radiocarbon‐dated specimens. Nine of these sequences originated from *M*. *arvalis* and have been previously reported (Baca et al., 2020). Three other dated sequences, that is, two from *M*. *arvalis* and one from the new mtDNA lineage, are reported here for the first time (Table S4). To increase the number of sequences from the newly reported mtDNA lineage for divergence dating analysis, we used the sequences of two specimens from layer G in Emine–Bair–Khosar dated to the post‐LGM period (Doan et al., 2018) and assigned them a sampling time of 15 cal ka BP. We used the Bayesian evaluation of temporal signal (BETS) (Duchene et al., 2020) to check whether sufficient temporal resolution is available within our dataset to calibrate the molecular clock. We compared the support for the four models; in two of them, we assigned real sampling times to the sequences (heterochronous analysis) and then used either strict clock or uncorrelated relaxed lognormal clock. In the two other models, we used the same sampling time (i.e., isochronous analysis) for all sequences and applied either strict clock or uncorrelated relaxed lognormal clock. We applied a constant population size tree prior for all analyses and a CTMC rate reference prior for the heterochronous datasets (Ferreira & Suchard, 2008). Each analysis was run for 20 million generations sampled every 2000 generations. We then estimated the log marginal likelihoods (MLE) of each model by using the generalized stepping‐stone (GSS) sampling approach (Baele et al., 2016). The MLE calculation comprised 50 path steps, each of which was run for 10^6^ iterations. We performed two replicates of each BEAST analysis. Convergence and stationarity were inspected in Tracer 1.7 (ESS > 200 for all parameters), and trees from the two runs were combined in *logcombiner* and summarized in *treeannotator*.

The mean divergence (*D_xy_*) between mtDNA lineages was calculated in MegaX (Kumar et al., 2018) by using the TrN substitution model with uniform rates across sites and 100 bootstrap replicates to determine the variance. Both transitions and transversions were included, and pairwise deletion was employed in case of missing data.

### 2.6 Radiocarbon dating

Three vole mandibles, one each from Obłazowa WE, Obłazowa 2, and Bacho Kiro Cave, were pre‐treated for radiocarbon dating at the Department of Human Evolution, Max Planck Institute for Evolutionary Anthropology (MPI‐EVA, Leipzig, Germany), following the protocol for <100 mg bone samples described in Fewlass et al. (2019). The quality of the collagen extracts was assessed based on the yield as a percentage of the original bone weight (minimum requirement, 1%). The elemental and isotopic ratios of the extracts (~0.5 mg) were measured on a Thermo Finnigan Flash elemental analyzer coupled to a Thermo Delta Plus XP isotope ratio mass spectrometer. If sufficient collagen was extracted, the collagen was graphitized using automated graphitization equipment (Wacker, Němec, et al., 2010) at the Laboratory of Ion Beam Physics, ETH‐Zurich (Switzerland) and dated on a MIni CArbon DAting System (MICADAS) accelerator mass spectrometer (Wacker, Bonani, et al., 2010) (Laboratory Code: ETH). If the extracted collagen yield was insufficient for graphitization, it was combusted to CO_2_ and measured directly using a gas interface system coupled to the gas ion source of the MICADAS (Wacker et al., 2013) following the protocol described in Fewlass et al. (2018, 2019). Radiocarbon dates were calibrated in OxCal v4.4 (Bronk Ramsey, 2009) by using the IntCal20 (Reimer et al., 2020) calibration curve.

## 3 RESULTS

Although we enriched our libraries for the whole mtDNAs, in numerous specimens, we encountered a mixture of at least two sequences spanning usually the first ca. 11 kb or, much less often, a larger portion of the mtDNA genome; thus, reliable consensus calling could not be achieved. The contaminating sequences were likely to be nuclear sequences of mitochondrial origin (*numts*). The presence of *numts* in mtDNA assemblies was previously reported for various *Microtus* species (Barbosa et al., 2018; Duckett et al., 2021; Triant & DeWoody, 2008), and their presence are especially expected in the case of the assemblies based on mtDNA‐enriched libraries. Therefore, we called consensus sequences only for mtDNA fragments where *numts* were not present, and this was either whole mtDNA, ca. 4.3 kb of mtDNA (12,001–16,267 according to NC_038176.1) or only the cytochrome b sequence (1143 bp) (Table S1). In the case of several specimens, the presence of second sequence prevented reliable consensus calling even for the cytochrome b fragment and such specimens were discarded (Table S1). We recovered at least the cytochrome *b* sequence of 22 specimens from the three investigated caves and the two common vole specimens from Poland. All the specimens yielded a damage pattern and length distribution of DNA molecules characteristic of ancient DNA (Table S1).

### 3.1 Phylogenetic analysis and divergence estimates

**FIGURE 1:**
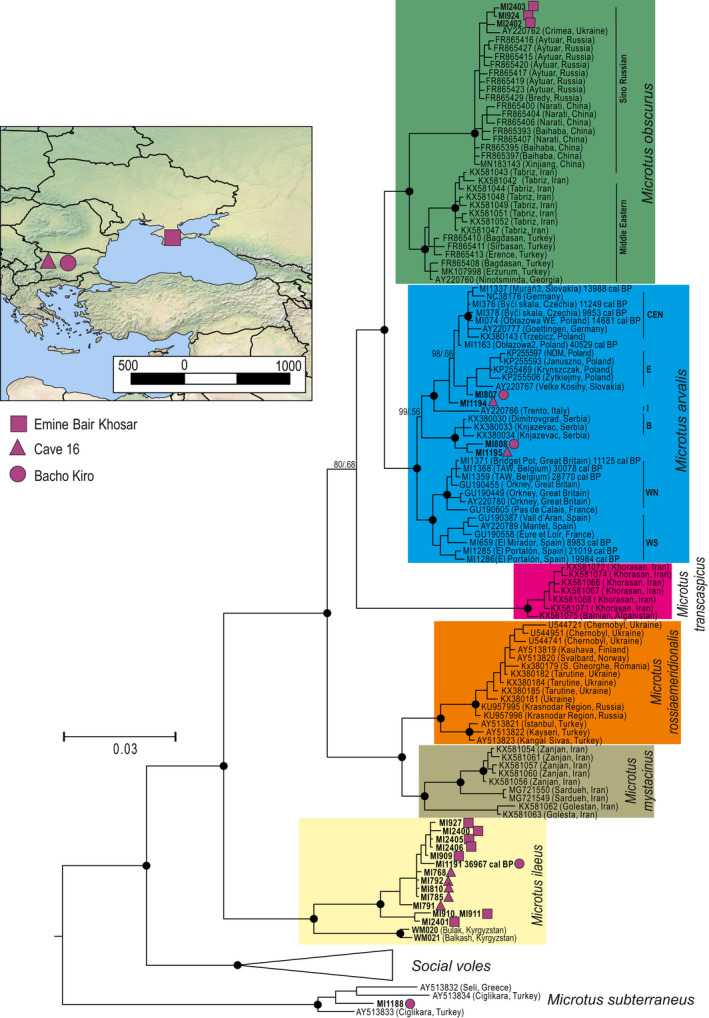
Bayesian phylogeny of the *Microtus* subgenus based on the mtDNA cytochrome *b* sequences (1143 bp). Dots at the main nodes indicate bootstrap support from IQtree and posterior probabilities from MrBayes greater than 90 and 0.95, respectively. Haplotypes obtained in this study are indicated in boldface. Violet symbols indicate the sampling site of the specimens as indicated in the inlet map. The median of the 95.4% calibrated range ages of the directly radiocarbon dated specimens are given in the labels

The phylogeny based on the mtDNA of cytochrome *b* recovered all main mtDNA lineages of grey voles with high support (Figures 1 and S1). One specimen from Bacho Kiro cave was revealed to be European pine vole (*M*. *subterraneus)*. Four specimens were classified as common vole (*M*. *arvalis)*, two from Bacho Kiro and two from Cave 16, another three specimens from Emine–Bair–Khosar cave were identified as *M*. *obscurus*. A total of 14 specimens, specifically, eight from Emine–Bair–Khosar, five from Cave 16, and one from Bacho Kiro, formed a previously unrecognized, highly supported lineage sister to the Tien Shan vole (*M*. *ilaeus*; Figures 1 and S1). The mean divergence (D_xy_) of this new lineage and the Tien Shan vole was 4.8% ± 0.6%, which is slightly higher than the D_xy_ between *M*. *arvalis* and *M*. *obscurus* (3.9% ± 0.5%) and similar to the D_xy_ of *M*. *mystacinus* and *M*. *rossiaemeridionalis* (4.8% ± 0.7%; Table S3). It was also much higher than divergence between *M*. *ilaeus* and *M*. *ilaeus igromovi* (2.6% ± 0.7%; based on 341‐bp fragment; Table S3).

### 3.2 Estimation of divergence times within the subgenus *Microtus*

**FIGURE 2:**
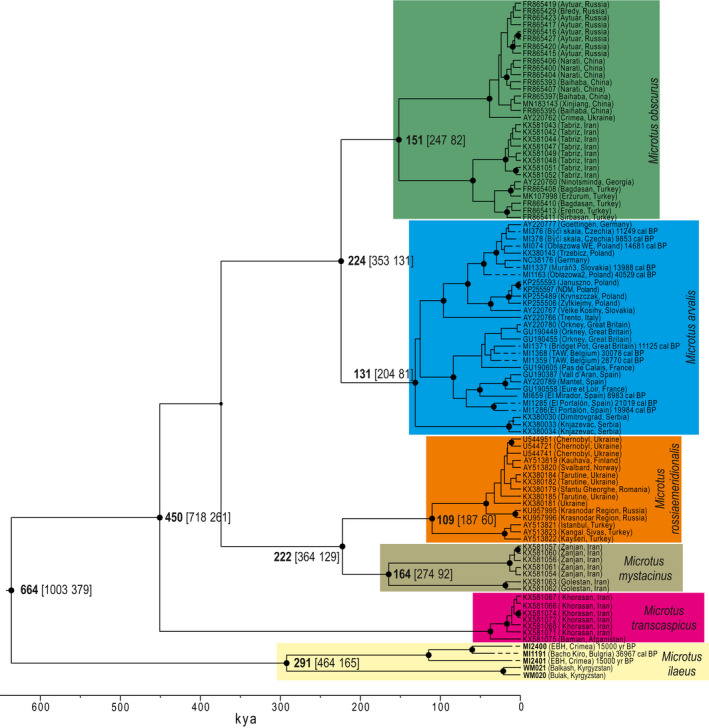
Maximum clade credibility phylogeny of the subgenus *Microtus*. The phylogeny was reconstructed in BEAST and is drawn to a timescale. Black dots indicate nodes with >0.95 posterior probability. The estimated divergence times with their 95% highest posterior density intervals are indicated next to the nodes leading to the main lineages. The median of the 95.4% calibrated range ages of the directly radiocarbon dated specimens are given in the labels

The three specimens pre‐treated for radiocarbon dating produced high‐quality collagen in terms of Carbon‐to‐Nitrogen ratio and yield (Table S4; van Klinken, 1999; Talamo et al., 2021). The resulting dates were consistent with the stratigraphic position of the specimens (Text S1) and used for calibration of the molecular clock. BETS analysis showed that our dataset contains sufficient temporal signal to calibrate the molecular clock. Amongst the models tested, the model with a strict molecular clock and correctly assigned sampling times produced the highest log MLE and it was highly supported (2lnBF = 23.54, 19.52, and 15.09 with respect to models with the correct sampling times and an uncorrelated relaxed clock, the model with no sampling times and a strict clock, and the model with no sampling times and an uncorrelated relaxed clock, respectively; Table S5). The validity of our dataset to estimate rates was further supported by the results of the date randomization test (Table S6). The maximum clade credibility tree obtained in BEAST revealed a topology similar to that obtained in MrBayes except for the position of *M*. *transcaspicus*, which was in a sister position with respect to *M*. *mystacinus*, *M*. *rossiaemeridionalis*, *M*. *obscurus*, and *M*. *arvalis*, while on the Bayesian tree it was a sister lineage to *M*. *obscurus* and *M*. *arvalis*. However, the position of this branch was recovered with low support in all approaches employed. The mean substitution rate of the mtDNA of cytochrome *b* was estimated for this dataset to be 1.25 × 10^−7^ substitutions/site/year^−1^ (95% highest posterior density interval (95% HPD): 7.24 × 10^−8^–1.82 × 10^−7^). The age of the crown of the *Microtus* subgenus was dated to ca. 0.66 Ma (95% HPD: 1.00–0.38 Ma). The divergence of *M*. *ilaeus* and the newly discovered lineage was estimated to be 0.29 Ma (95% HPD: 0.46–0.16 Ma), which is slightly earlier than the divergence of *M*. *arvalis* and *M*. *obscurus* (0.22 Ma (95% HPD: 0.36–0.13 Ma)) and the divergence of *M*. *mystacinus* and *M*. *rossiaemeridionalis*, (0.22 Ma (95% HPD: 0.36–0.13 Ma) (Figure 2).

### 3.3 Morphometric features of the new mtDNA lineage

**FIGURE 3:**
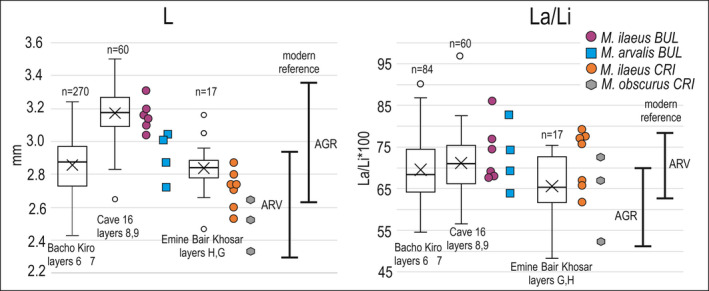
Morphometric characteristics of the newly identified mtDNA lineage of *Microtus ilaeus*. The boxplots summarize the variations in length (L) and La/Li index of the arvaloid m1s from the three studied sites. L is given according to Nadachowski (1984b) and Popov (2000). The box extends from 1st to 3rd quartile, while the whiskers extend to the data points within 1.5 times the interquartile range (IQR) from the ends of the box, values outside this range are denoted with circles. Vertical lines denote medians, whilst crosses denote mean values. The colored symbols represent individual measurements of specimens assigned to species using mtDNA sequences. The reference ranges for *M*. *arvalis* (ARV) and *M*. *agrestis* (AGR) are based on modern specimens according to Nadachowski (1984a)

**FIGURE 4:**
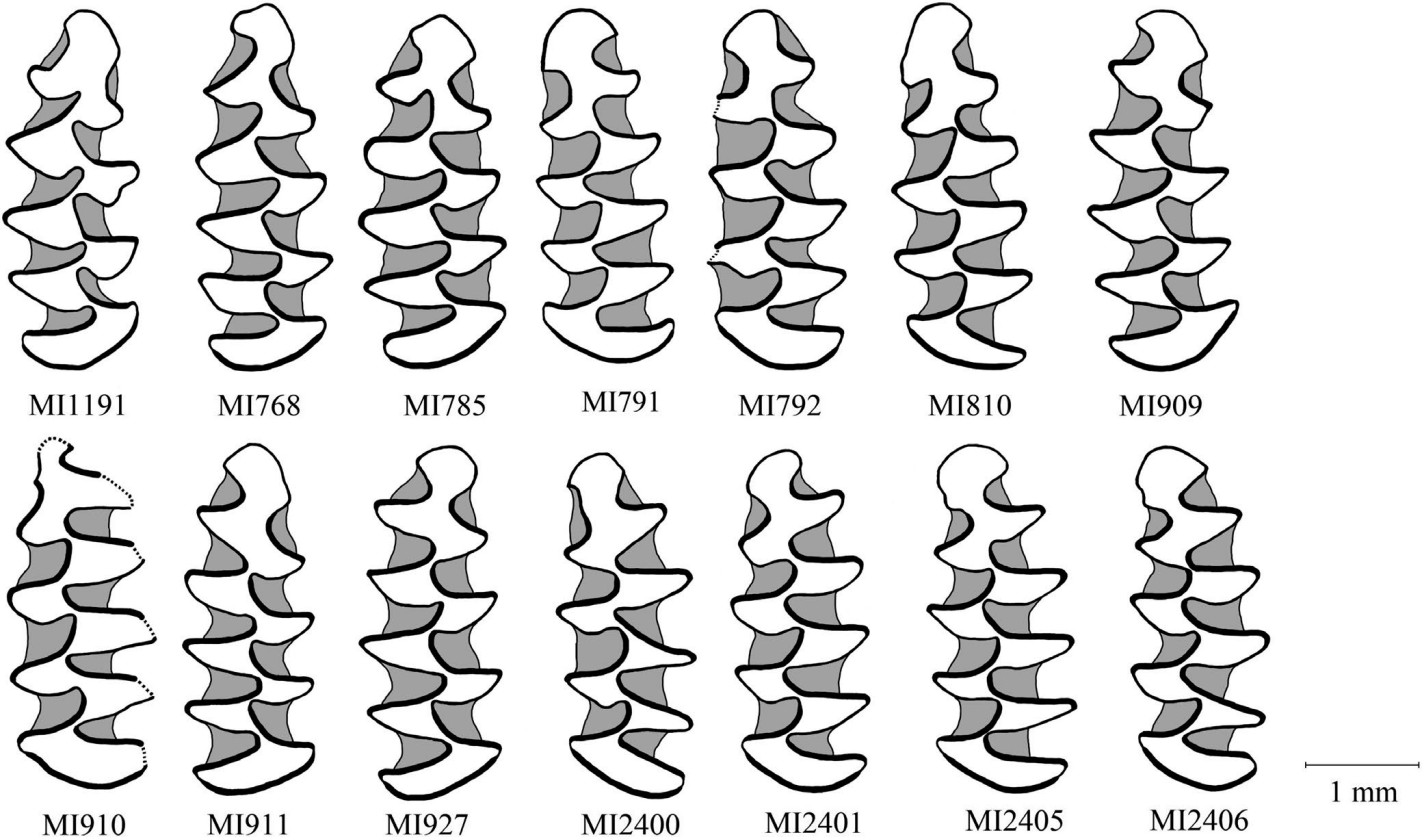
Drawings of the occlusal surface of the first lower molars (m1) of specimens belonging to the newly identified lineage of *Microtus ilaeus*. MI1191: Bacho Kiro cave, layer 7/6b; MI768–MI810: Cave 16, layer 9; MI909–MI927: Emine–Bair–Khosar cave, layer H; MI2400, MI 2401: Emine–Bair–Khosar cave, layer G; MI2405, MI2406: Emine–Bair–Khosar cave, layer H (Table S7)

Measurements were performed on all 23 specimens that yielded mtDNA sequences. We also calculated the La/Li index of subset specimens from Bacho Kiro and Cave 16 because this parameter has not been reported in original publications describing the assemblages of small mammals from these sites (Nadachowski, 1984b; Popov, 2000). The tooth size of specimens from the newly identified lineage was between 2.53 and 3.31 mm  ± *SD*, 2.92 ± 0.24; Figure 3; Table S7). The teeth of specimens from Bulgaria were significantly larger ($\bar{x}$ *L* = 3.16, *n* = 6) than those from Crimea ($\bar{x}$ *L* = 2.71, *n* = 7; *T*‐test, *p* < .005; Figure 3). In Bulgaria, specimens from the new lineage were larger than those of *M*. *arvalis* and exceeded the size range of *M*. *agrestis* (Figure 3), but this result must be treated with caution because only very small number of individuals were measured. The La/Li index varied between 61.4 and 85.7 ($\bar{x}$ ± SD, 72.55 ± 6.61; *n* = 14) and were within the range recorded for *M*. *arvalis*, that is, generally exceeded 65.0 (Nadachowski, 1984a; Figure 3; Table S7). The La/Li index for a random subset of specimens from Bacho Kiro ($\bar{x}$ ± SD, 67.98 ± 7.41; *n* = 84) and Cave 16 ($\bar{x}$ ± SD, 71.18 ± 6.75; *n* = 60) also yielded values in the range of species from the *arvalis* group (Figure 3). The anterioconid complex of *M*. *ilaeus*, especially the anterior cap, was highly variable, and no morphological features that could clearly distinguish *M*. *ilaeus* from *M*. *arvalis* and/or *M*. *obscurus* were found (Figure 4).

## 4 DISCUSSION

### 4.1 Characterization of the newly discovered lineage

Investigation of ancient DNA from Late Pleistocene vole specimens from three cave sites located in the Black Sea area revealed the existence of a *Microtus* species previously unknown in Europe and representing a divergent mtDNA lineage of the Tien Shan vole (*M*. *ilaeus*).

The known range of the newly characterized lineage is, at this point, limited to area around the Black Sea, over 2000 km from the range of the extant *M*. *ilaeus igromovi*, which is currently limited to the small area east of the Aral Sea and more than 3500 km from the main species range in Central Asia. However, given the early divergence of this species in the history of the subgenus, it is probable that the ranges of this species were larger earlier in the Pleistocene. In both localities in Bulgaria, *M*. *ilaeus* specimens belonging to the new mtDNA lineage co‐occurred in the same layers with *M*. *arvalis*; in Crimea, *M*. *ilaeus* and *M*. *obscurus* were found together in layer G. Although we cannot be certain, the population of *M*. *ilaeus* appears to have lived with *M*. *arvalis* and *M*. *obscurus* in sympatry.

Investigation of tooth morphology revealed no clear differences between the newly discovered lineage and other species from *Microtus* s. str. (Figure 4, Table S7). The size of the teeth (L) of the new species was highly variable but remained within the range of tooth sizes reported for other species from the subgenus (Mahmoudi, Kryštufek, et al., 2017). Differences in tooth size between *M*. *ilaeus* from Bulgaria and Crimea and *M*. *ilaeus* and *M*. *arvalis* from Bulgaria may reflect partitioning into different ecological niches; the current data, however, are too limited to build any definite conclusions.

The difference in size between *M*. *arvalis* and *M*. *ilaeus* from Bulgaria may have, however, some implications for understanding of paleontological small mammal assemblages from the area. Amongst rodents from the late Middle and Late Pleistocene of Bulgaria, voles with the arvaloid m1 morphology were found in 11 sites (Popov, 2018). These voles are most often referred to as *Microtus* ex gr. *arvalis*/*agrestis* (Nadachowski, 1984b; Popov, 2000, 2018; Popov & Marinska, 2007) or *Microtus* ex gr. *arvalis* (Ivanova et al., 2016; Popov, 1994). Because of the large variability and range of the m1 length of specimens with *arvaloid* morphology from Bacho Kiro and Cave 16, these specimens were previously suggested to belong to two species: the smaller *M*. *arvalis* and the larger *M*. *agrestis* (Nadachowski, 1984b; Popov, 2000). Considering the genetic and morphometric data obtained in this study, these species may be considered to actually be two species from the *arvalis* group, namely, the smaller *M*. *arvalis* and the larger *M*. *ilaeus*, not *M*. *agrestis*, especially because the latter species does not occur in Bulgaria today (Shenbrot & Krasnov, 2005) and was not indisputably found in the Late Pleistocene (Popov, 2018).

In contrast to other species of the subgenus *Microtus*, the newly described mtDNA lineage of *M*. *ilaeus* most probably did not survive into the present time; however, the exact timing and causes of its extinction remain unclear. Specimens from Cave 16 were obtained from layers below the Campanian Ignimbrite tephra dated to ca. 40 ka (Giaccio et al., 2008; Popov, 2018), which is slightly older than the age of the specimen from Bacho Kiro. The distribution of large (*L* > 3.1 mm) teeth at both Bulgarian sites, which were previously considered *M*. *agrestis* and now may be attributed to *M*. *ilaeus*, may provide more detailed insight into the chronology of this form. In Cave 16, such large teeth were found nearly exclusively in layers 9 and 8, both of which date to over 40 kya (Popov, 2000; Text S1). In Bacho Kiro, the same‐size teeth occurred in multiple layers, with a noticeable peaks in layers 13 and 6b–7, and disappeared in layer 3. The new radiocarbon chronology of the site suggests that layer 13 dates to more than 51 ky BP, while the layers 6b and 7 to 43–36 ka cal BP (Fewlass et al., 2020). There are no radiocarbon dates from layer 3 but based on small mammal assemblage it was dated to the LGM (Kozłowski, 1982; Nadachowski, 1984b). Specimens belonging to the new mtDNA lineage from Emine–Bair–Khosar were distributed across layers H and G between depths of 4.8 and 1.9 m. Layer H is dated to the older part of MIS 3 (between >50 and 36 ka cal BP) (Ridush et al., 2013). The youngest specimens come from the uppermost part of layer G. This layer is attributed to MIS 2, most probably the post‐LGM or even the Late Glacial period (Ridush et al., 2013). These findings suggest that the extinction of the newly described mtDNA lineage may have been caused by environmental and climatic changes associated with the Pleniglacial to the Late Glacial or the Late Glacial to the Holocene transitions.

### 4.2 Molecular rates and dating of divergences within subgenus *Microtus*

The investigated dataset enabled the calibration of the molecular clock and estimation of the divergence time of the newly identified mtDNA lineage and extant *M*. *ilaeus*, as well as other species and forms within subgenus *Microtus*. The estimated substitution rate of the mtDNA of cytochrome *b* (1.25 × 10^−7^ substitution/site/year^−1^) was ca. three times slower than the rates estimated previously for *M*. *arvalis* (3.27 × 10^−7^) (Martínková et al., 2013) and *M*. *agrestis* (3.89 × 10^−7^; Herman & Searle, 2011), likely because of the time dependence of the molecular rates of mtDNA. Estimates of evolutionary rates are known to scale negatively with the age of calibration (Ho et al., 2011; Molak & Ho, 2015). In previous studies, very recent calibration points were used to estimate rates; in the case of *M*. *arvalis*, these points were sequences from specimens radiocarbon‐dated to no more than 6 kya. In the case of *M*. *agrestis*, the calibration points employed were biogeographic events younger than 10 kya. The substitution rates obtained from such calibration are more suitable for the estimation of recent, intraspecific rather than interspecific events. In the present work, we used calibration specimens dated up to 40 kya to estimate interspecific divergence times. Consequently, our estimates were nearly two times older than those proposed by Mahmoudi, Darvish, et al. (2017), who based their calculations on the substitution rate of Martínková et al. (2013). Our estimates are also nearly two times younger than the estimates based on fossil calibration (Thanou et al., 2020). Our estimate of the age of the crown of the *Microtus* subgenus (0.66 Ma (95% HPD 1.00–0.38 Ma)) was remarkably similar to that obtained by Bannikova et al. (2010) (0.54 ± 0.13 Ma), who used fossil calibration (basal radiation of the *Microtus* genus set to 2.2 ± 0.2 Ma) to calibrate the molecular clock for the mtDNA cytochrome *b* phylogeny but accounted for the rate decay. These authors assumed that transversions at the third‐codon position are only slightly affected by rate decay phenomena and used the relationship between the divergence calculated using all positions and that calculated using only transversions at the third‐codon position to transform node heights (Bannikova et al., 2010). The obtained estimation is also highly consistent with the first appearance of the *M*. *arvalis* group in fossil record (MIS 14–11; 0.60–0.45 Ma). Although, the distinction between species from the arvalis group and *M*. *agrestis* in fossil record may be difficult as exemplified by our study, the appearance of arvalis group around 0.60–0.45 Ma was reported independently by multiple authors (Berto et al., 2021; Fejfar, 1965; Kolfschoten, 1990; Maul & Markova, 2007). Among others, Berto et al. (2021) used morphological features to distinguish *M*. *arvalis* from *M*. *agrestis* and provided the most compelling evidence for presence of the former in the Central Europe during at least MIS 11 and most probably during MIS 14. The consistency of estimates based on distinct calibration methods and fossil record indicates that the estimated divergence times obtained in this work may be close to the real values.

### 4.3 Taxonomic status of the newly identified lineage

The genetic divergence of the extant *M*. *ilaeus* and the newly discovered mtDNA lineage is similar to that of *M*. *arvalis* and *M*. *obscurus* and that of *M*. *mystacinus* sensu stricto and *M*. *rossiaemeridionalis*; this finding allows for various interpretations. On the one hand, the latter forms seem to be on the verge of speciation (Barbosa et al., 2018), and their taxonomic status is a matter of ongoing debate (Mahmoudi et al., 2014). Recent studies on the reproductive isolation and hybrid sterility of the *M*. *mystacinus* group (i.e., *M*. *mystacinus*, *M*. *rossiaemeridionalis*, and *M*. *kermanensis*) demonstrated the interspecies hybrid sterility of males and most females (Bikchurina et al., 2021). Therefore, the taxonomic forms identified above should be considered valid species despite their low genetic divergence of only 4%–5%. The mtDNA divergence between the newly identified lineage and *M*. *ilaeus* is also much higher than between the latter and *M*. *ilaeus igromovi*, which are considered subspecies (Figure S1; Golenishchev et al., 2019). This reinforces the interpretation that the newly identified lineage represents a distinct species. On the other hand, Allen et al. (2020) suggested that interspecific reproductive isolation in mammals appears when *cytb* DNA sequences differ by over 7.2%. Because of these differences in interpretation of mtDNA diversity, we do not postulate taxonomic changes in the *M*. *arvalis* species group. Further accumulation of genomic and morphological data will provide a better understanding of the evolutionary history of this lineage.

Overall, our ancient DNA study revealed the existence of a previously unknown, highly divergent population of the Tien Shan vole (*M*. *ilaeus*) in the area adjacent to the Black Sea, over 2000 km from the extant range of this species. The mtDNA divergence of the European population and the extant form was high, similar to other forms within the *M*. *arvalis* species group, which are considered different species or on the verge of speciation. This population lived in sympatry with other grey voles and eventually became extinct after the LGM, likely as a result of climate changes in the Pleniglacial to the Late Glacial or the Holocene transition. This study indicates that species of the *Microtus* subgenera may have been more diverse during the Late Pleistocene than previously established and highlights the use of ancient DNA as an important tool to decipher this diversity.

## CONFLICT OF INTEREST

The authors declare that they have no conflict of interest.

## AUTHOR CONTRIBUTIONS

**Mateusz Baca:** Conceptualization (equal); Data curation (equal); Formal analysis (lead); Funding acquisition (lead); Investigation (lead); Supervision (equal); Visualization (lead); Writing‐original draft (lead); Writing‐review & editing (equal). **Danijela Popović:** Data curation (equal); Formal analysis (equal); Investigation (equal); Project administration (equal); Writing‐review & editing (equal). **Anna Lemanik:** Data curation (equal); Investigation (equal); Writing‐review & editing (equal). **Helen Fewlass:** Formal analysis (supporting); Investigation (supporting); Writing‐review & editing (equal). **Sahra Talamo:** Investigation (supporting); Supervision (supporting); Writing‐review & editing (equal). **Bogdan Ridush:** Resources (equal); Writing‐review & editing (equal). **Jan Zima:** Resources (equal). **Vasil Popov:** Resources (equal); Writing‐review & editing (equal). **Adam Nadachowski:** Conceptualization (equal); Funding acquisition (supporting); Resources (equal); Writing‐original draft (equal).

## Supporting information

Supplementary Material

Table S1‐S7

## Data Availability

DNA sequences were deposited in GenBank under accession no. MZ362409–MZ362424; MZ438664–MZ438673.
