# Supplementary material for: The Tien Shan vole (Microtus ilaeus; Rodentia: Cricetidae) as a new species in the Late Pleistocene of Europe
Source: Ecol Evol. 2021 Nov 4;11(22):16113–25. doi: 10.1002/ece3.8289 (PMC8601874; doi:10.1002/ece3.8289)
Supplement: Supplementary file 1 — Supplementary Material [file ECE3-11-16113-s002.docx]

**The Tien Shan vole (*Microtus ilaeus*; Rodentia: Cricetidae) as a new species in the Late Pleistocene of Europe**

Mateusz Baca^1^, Danijela Popović^1^, Anna Lemanik^2^, Helen Fewlass^3^, Sahra Talamo^3,4^, Jan Zima^5†^, Bogdan Ridush^6^, Vasil Popov^7^, Adam Nadachowski^2^

^1^ Centre of New Technologies, University of Warsaw, S. Banacha 2c, 02-106 Warszawa, Poland

^2^ Institute of Systematics and Evolution of Animals, Polish Academy of Sciences, Sławkowska 17, 31-016 Kraków, Poland

^3^ Department of Human Evolution, Max Planck Institute for Evolutionary Anthropology, Deutscher Platz 6, D-04103 Leipzig, Germany

^4^Department of Chemistry G. Ciamician, University of Bologna, Via Selmi 2, 40126 Bologna, Italy

^5^ Institute of Vertebrate Biology, Academy of Sciences of Czech Republic, Kvĕtná 8, 603 65 Brno, Czech Republic

^6^ Department of Physical Geography, Geomorphology and Paleogeography, Yuriy Fedkovych Chernivtsi National University, Kotsubynskogo 2, 58012 Chernivtsi, Ukraine.

^7^ Institute of Biodiversity and Ecosystem Research, Bulgarian Academy of Sciences, 2 Gagarin Street, 1113 Sophia, Bulgaria

^†^deceased

# Supplementary Figures

Figure S1. Bayesian phylogeny of subgenus Microtus based on 341 bp fragment of mtDNA cytochrome b. Dots at nodes indicate posterior probabilities from MrBayes greater than 0.95. For the main nodes posterior probabilities lower than 0.95 are given. Haplotypes obtained in this study are indicated in boldface.

# Description of paleontological sites

## Bacho Kiro Cave, Praebalkan region, Dryanovo, Bulgaria

Bacho Kiro Cave (25°25'46.27"E, 42°57'2.27"N, 327 m a.s.l.) was first excavated in 1938 during the American expedition of D. Garrot (1939), with the cooperation of R. Popov. New research was conducted by Regional Museum of Gabrovo (Bulgaria) and Institute of Archaeology of Jagiellonian University (Poland) during the years 1971-1975. The results were presented in a book devoted to various aspects of archaeological, stratigraphic, and palaeontological studies undertaken in the site (Kozłowski, 1982). From the lithological and stratigraphical point of view, the Bacho Kiro deposits were divided into three sedimentation series and 21 layers corresponding to the interval between MIS 4 and MIS 1, with hiatuses. Most of them yielded numerous faunal remains (108 species). New excavations carried out in recent years (2015-2018) by the National Archaeological Institute with Museum in Sofia and the Department of Human Evolution at the Max Planck Institute for Evolutionary Anthropology in Lipzig have provided spectacular discoveries of the oldest representatives of *Homo sapiens* in Europe (e.g., Hublin *et al.*, 2020), including numerous materials of small mammals which, however, have not yet been analysed in detail. The recent excavations resulted also in the refined stratigraphy of the site, based on numerous radiocarbon dates (Fewlass *et al*, 2020). The research on rodent fossil remains carried out in this study comes exclusively from the excavations undertaken by the Polish-Bulgarian team in the early ‘70s. The rodents from these studies were initially studied by Kowalski and Nadachowski (1982), and later Nadachowski (1984) described the remains of *Arvicolinae* in detail.

### References

Garrot D. 1939. Excavations in the cave of Bacho Kiro, *American School of Prehistoric* *Researches – Bulletin* 15: 46–70.

Fewlass H, Talamo S, Wacker L, Kromer B, Tuna T, Fagault Y, Bard E, McPherron SP, Aldeias V, Maria R, Martisius NL. 2020. A 14 C chronology for the Middle to Upper Palaeolithic transition at Bacho Kiro Cave, Bulgaria, *Nature ecology and evolution* 4(6): 794–801.

Hublin J-J, Sirakov N, Aldeias V, Bailey S, Bard E, Delvigne V, Endarova E, Fagault Y, Fewlass H, Hajdinjak M, Kromer B, Krumov I, Marreiros J, Martisius NL, Paskulin L, Sinet-Mathiot V, Meyer M, Pääbo S, Popov V, Rezek Z, Sirakova S, Skinner MM, Smith GM, Spasov R, Talamo S, Tuna T, Wacker L, Welker F, Wilcke A, Zahariev N, McPherron SP, Tsanova T. 2020. Initial Upper Palaeolithic *Homo sapiens* from Bacho Kiro, Bulgaria, *Nature*, 581: 299–315.

Kowalski K, Nadachowski A. 1982. Animal remains - Rodentia. In: Kozłowski JK, ed. Excavations in the Bacho Kiro Cave (Bulgaria) - Final report. Państwowe Wydawnictwo Naukowe, Warszawa, 45–51.

Kozłowski JK. (Ed.). 1982. Excavation in the Bacho Kiro Cave. Final raport. Warszawa. Państwowe Wydawnictwo Naukowe.

Nadachowski A. 1984. Morphomertic variability of dentition of the Late Pleistocene voles (Arvicolidae, Rodentia) from Bacho Kiro Cave (Bulgaria), *Acta Zoologica Cracoviensia*, 27(9): 149–176.

## Cave 16, Praebalkan region, Karlukovo, Bulgaria

Cave 16 (43°10 37.31"N, 24°4’20.33"E, 234 m a.s.l.) is a large rock niche, half-filled with sediments. At the entrance, the sediments were exposed by erosion, creating a well-stratified profile over 5 m thick. During pilot archaeological excavations in 1984 - 1993, the profile was cleared, and its stratigraphy was studied. 15 layers have been identified (Ferrier, 1994, Popov, 1994, 2000). Both now and in the recent geological past, the cave is and has been a nesting place for many petrophilous birds, including owls. This is evidenced by numerous owl pellets on the modern surface of the sediments and the high accumulation of bones in the deposits from layer 15 upwards. Based on the ecological appearance of the stratigraphic small mammal assemblages, the layers form two distinct groups - upper (layers 9 - 1) and lower (layers 15 - 10). Among the layers forming the upper series, particular attention is paid to layer 7, which consists of volcanic ash. It has been found to result from one of the most powerful volcanic eruptions in southern Italy, known as the Campanian Ignimbrite (CI), dating from ca. 40,000 years BP (Giaccio *et al*., 2008). The assemblages from the lower part of the profile are characterized by a high proportion of forest and mesophilic species, as well as thermophilic sub-Mediterranean elements; the inhabitants of the steppes have a low share. Quantitative paleoecological reconstructions (Popov, 2014, 2018) suggest that during the formation of the lower part of the sediment sequence (layers 14-10) the temperatures were similar to the modern ones. However, the humidity for most of this period was higher than now. The climate and vegetation were comparable to those in the mountains of Central Europe. The small mammal assemblages at the top of the profile show a high percentage of species characteristic of open landscapes. It can be said that the layers of the upper part of the profile (layers 9-1) were formed in a cooler climate, especially in the layers deposited before and after the volcanic ash. Winter temperatures were about 10 degrees lower than today. The seasonal temperature contrast was significant. The rainfall was less than today. In the age's context of volcanic ash and based on both the ecological appearance of small mammals and quantitative climatic reconstructions, it can be said that layers 14 - 10, showing a milder climate, are most likely to be referred to the end of the last interglacial period; layers 10 - 8 reflect the progressive cooling during the early Last Glacial (MIS 4 - 3), corresponding to the time interval between 90,000 and 50,000 years BP; layers 6 - 1 correlate with the Last Glacial Maximum (LGM).

In this context, special attention should be paid to layer 9. It has a significant thickness, as it consists of huge limestone blocks, the result of erosion of the walls and the arch of the rock niche. Given that the niche is wide open, it can be assumed that this type of sedimentation is the result of significant seasonal climatic amplitudes. The climate was probably very continental. This is confirmed by the dominance of a typical steppe dweller as *Lagurus lagurus*, which only in this layer has a relative abundance exceeding that of *Microtus* ex gr. *arvalis* (Popov, 2018, Fig. 8). Throughout the sequence, *Microtus* ex gr. *arvalis* shows well-defined morphological changes, expressed in an increase in the size of m1 from layer 14 upwards, reaching the largest dimensions in layers 9 and 8. Then the size decreases again (Fig. S1). In the light of the new data, it can be assumed that these changes reflect a gradual increase in the relative abundance of the newly described form belonging to *M. ilaeus* group, which becomes dominant in layers 9 and 8. Given the climatic interpretation presented above, it can be assumed that the continental climate during the deposition of layers 9 and 8 has favored the westward range expansion of Central Asian species such as *Lagurus lagurus* and *Microtus* ex gr. *ilaeus.*

Figure S2. Box plots summarising the variations in length of arvaloid m1 teeth in subsequent layers of Cave 16. The box extends from 1^st^ to 3^rd^ quartile, while the whiskers extend to the data points within 1.5 times the interquartile range (IQR) from the ends of the box, values outside this range are denoted with circles. Vertical lines within boxes denote medians, whilst crosses denote mean values. Due to the small number of specimens, measurements from layers 6 and 7 are presented together.

### References

Ferrier C. 1994. Le context environmental du peuplement paleolithique de Bulgarie du Nort. Le karst de Karlukovo et ses depots. Thesis. L'Universite Bordeuax I.

Giaccio B, Isaia R, Fedele FG, Di Canzio E, Hoffecker JF, Ronchitelli A, Sinitsyn AA, Anikovich M, Lisitsyn SN, Popov VV. 2008. The Campanian Ignimbrite and Codola tephra layers: two temporal/stratigraphic markers for the Early Upper Palaeolithic in southern Italy and eastern Europe, *Journal of Volcanology and Geothermal Research*, 177: 208–226.

Popov VV. 1994. Quaternary small mammals from deposits in Temnata - Prochodna Cave system. In: Kozlowski JK, Laville H, Ginter B, eds. Temnata Cave, Excavations in Karlukovo Karst Area, Bulgaria Krakow: Jagellonian University Press, 1(2) 11–53).

Popov VV. 2000. The small mammals (Mammalia: Insectivora, Chiroptera, Lagomorpha, Rodentia) from Cave 16 (North Bulgaria) and the paleoenvironmental changes during the Late Pleistocene. In: Ginter B, Kozlowski JK, Laville K, eds. Temnata Cave. Excavations in Karlukovo Karst Area, Bulgaria, Krakow, Jagellonian University Press 2(1): 13–65.

Popov VV, Di Canzio E, Giaccio B. 2014. Late Quaternary Small Mammals and Paleotemperatures in Bulgaria and Italy, *Acta Zoologica Bulgarica* 66: 89–108.

Popov VV. 2018. Pliocene-Quaternary Small Mammals (Eulipotyphla, Chiroptera, Lagomorpha, Rodentia) in Bulgaria: Biostratigraphy, Paleoecology, and Evolution. In: Huard G, Gareau J, eds. The Pleistocene: Geography, Geology, and Fauna. Nova Science Publishers, 109–235.

### Emine-Bair-Khosar Cave, Chatyrdag Plateau, Crimea, Ukraine

Emine-Bair-Khosar Cave (44°48'4.65"N, 34°17'21.99"E) is known as a huge trap-cave on the Chatyrdag Plateau (Crimean Mountains, Ukraine), at the 982 m a.s.l. Totally in the cave, there are 11 sites (Ba1, Ba2, Bb, Bc, Bd, Be, Bf, Bg, Bh, Bi, Bj) with subfossil bone remains, but only two of them (Ba2 and Bc) are stratified and represent a continuous section from the end of Middle Pleistocene to Holocene. The Bb site contains mainly bone breccia, inside the debris cone, under the entrance pit (Vremir & Ridush, 2005; Ridush *et al.*, 2013, 2018). The bone sites are of few taphonomic types. Big and middle-size mammals’ remains accumulated due to animals’ falling into the wide (7-8 m) and deep (13 m) pit-entrance. Small mammals, reptiles, amphibians, and small birds also accumulated due to birds of prey nesting in the cave, close to the entrance. The first bone site in the cave, mainly with large carnivores, was found at some distance from the pit at the beginning of the 1960s by Bachynsky and Dublyasky (1966). In the 1980s, A. Kozlov discovered two new bones site, which exploration started only in 2000 by Vremir and Ridush (2002) when new sites were discovered (Vremir & Ridush, 2005). From 2005 till 2013, exploration continued by Yuriy Fedkovych Chernivtsi University with the participation of Wroclaw University (since 2008) (Ridush *et al.,* 2013).

The main section at the Ba2 site includes several stratigraphic units: A, B, C, D, and E refer to Holocene and contain mainly small vertebrates. Unit F correlates to the Late Glacial (MIS-2) and contains some large mammals’ bones. Unit G suggested to be accumulated during Late Pleniglacial (MIS-2) and contain more numerous large mammals remains. Unit H is the richest in large bones and corresponds with the Middle Pleniglacial (MIS 3). Unit I belong to Eemian interstadial (MIS 5e). Some cold episodes of the Late Pleistocene are not represented in the sequence, probably due to a snow-ice plug in the entrance pit. The Bc site that continues the sequence was dated after *Mammuthus intermedius* approximately to MIS 7 (Ridush *et al.*, 2018). The trap type of accumulation caused nice preservation of bones, skulls and partial skeletons that enabled their morphometric studies (van Asperen *et al.*, 2012; Ratajczak et al., 2016). Because of the low and stable temperatures in the cave (around 5ºC), we can note quite good preservation of DNA, which allowed DNA isolation from numerous samples of different species (Stankovic *et al.*, 2011; Doan *et al.*, 2018; Niedziałkowska *et al*., 2020).

### References

Asperen EN, van, Stefaniak K, Proskurnyak I, Ridush B. 2012. Equids from Emine-Bair-Khosar Cave (Crimea, Ukraine): co-occurrence of the stenonid *Equus hydruntinus* and the caballoid *E. ferus* latipes based on skull and postcranial remains, *Palaeontologia Electronica* 15: 1-28.

Bachynsky GA, Dublyansky VN, 1966. Novi znakhidky vykopnykh khrebetnykh v hlybynnykh karstovykh porozhnynakh Hirskogo Krymu. In: Voistvenskyi, MA, Kistiakivkyi, OB, Abelentsev, VI, Sokur, IT., Gizenko, OI, Girenko, LL eds, Ekologia Ta Istoria Fauny Khrebetnykh Ukrainy. Naukova Dumka, Kyiv, 110–116.

Doan K, Mackiewicz P, Sandoval-Castellanos E, Stefaniak K, Ridush B, Dalén L, Wȩgleński P, Stankovic A. 2018. The history of Crimean red deer population and Cervus phylogeography in Eurasia, *Zoological Journal of the Linnean Society* 183: 208–225.

Niedziałkowska M, Doan K, Górny M, Sykut M, Stefaniak K, Piotrowska N, Jędrzejewska B, Ridush B, Pawełczyk S, Mackiewicz P, Schmölcke U, Kosintsev P, Makowiecki D, Charniauski M, Krasnodębski D, Rannamäe E, Saarma U, Arakelyan M, Manaseryan N, Titov VV, Hulva P, Bălășescu A, Fyfe R, Woodbridge J, Trantalidou K, Dimitrijević V, Kovalchuk O, Wilczyński J, Obadă T, Lipecki G, Arabey A, Stanković A. 2021. Winter temperature and forest cover have shaped red deer distribution in Europe and the Ural Mountains since the Late Pleistocene, *Journal of Biogeography* 48: 147–159.

Ratajczak U, Shpansky AV, Malikov DG, Stefaniak K, Nadachowski A, Wojtal P, Ridush B, Krakhmalnaya TV, Stepanchuk V, Mackiewicz P. 2016. Quaternary skulls of the saiga antelope from Eastern Europe and Siberia: Saiga borealis versus Saiga tatarica – One species or two? *Quaternary* *International* 420: 329–347.

Ridush B, Stefaniak K, Socha P, Proskurnyak Y, Marciszak A, Vremir M, Nadachowski A, 2013. Emine-Bair-Khosar Cave in the Crimea, a huge bone accumulation of Late Pleistocene fauna, *Quaternary* *International* 284: 151–160.

Ridush B, Stefaniak K, Nadachowski A, Ratajczak U, Socha P, Popiuk Y, Ridush O, Nykolyn O. 2018. Pleistocene fauna of the Emine-Bair-Khosar Cave (Crimea, Ukraine): new data. In: Zupan Hajna N, Mihevc A, Năpăruș-Aljančič M, eds. Quaternary Stratigraphy in Karst and Cave Sediments. INQUA Section on European Quaternary Stratigraphy. Karst Research Institute SRC SASA, Postojna, Slovenia, 57–59.

Stankovic A, Doan K, Mackiewicz P, Ridush B, Baca M, Gromadka R, Socha P, Węgleński P, Nadachowski A, Stefaniak K. 2011. First ancient DNA sequences of the Late Pleistocene red deer (*Cervus elaphus*) from the Crimea, Ukraine, *Quaternary* *International* 245: 262–267.

Vremir M, Ridush B. 2002. Recent palaeontological investigations in some caves of the Crimean mountain-range ( SE Ukraine ), *Theoretical and Applied Karstology* 15: 125–132.

Vremir MM, Ridush B, 2005. The Emine-Bair-Khosar “Mega Trap” (Ukraine), *Mitteilungen der Kommission für Quartärforschung Österreichischen Akademie der Wissenschaften* 235–239.

## Obłazowa Cave (western entrance), Western Carpathians, Nowa Biała, Poland

Obłazowa Cave (49°25’48”N, 20°09’36”E) is located in the south-western part of the Obłazowa Rock in the Pieniny Klippen Belt, Western Carpathians, Poland. The site yielded a number of important archaeological inventories, the oldest anatomically modern human (*Homo sapiens)* remains in Poland as well as abundant and diversified fauna discovered in a series of sediments divided into 21 layers (e.g. Valde-Nowak et al. 1987, 2003; Trinkaus et al. 2014; Valde-Nowak and Nadachowski 2014). The western entrance to the cave (Obłazowa cave WE) was discovered in 2009 and intensively explored in years 2015-2016. A 2 x 2 m trench was situated next to the rocky wall directed to the west which forms an overhang. The sediments were located close to the limestone wall, under the overhang, and partly on the slope. Its depth reached ca. 60-70 cm. The profile consists of three layers but remains of fauna was found only in layers II and III. At the top of layer II the Late Magdalenian inventories and fire place were discovered (Valde-Nowak et al. 2018). An accumulation of sediments took placed in the end of Late Pleistocene (GS-2a and GI-1e), ca. 16,1-14,0 ka cal BP (Nadachowski and Valde-Nowak, 2015; Lemanik et al., 2020).

**References**

Lemanik A, Baca M, Wertz K, Socha P, Popović D, Tomek T, Lipecki G, Kraszewska A, Miękina B, Żeromska A, Pereswiet-Soltan A, Szyndlar Z, Cieśla M, Valde-Nowak P, Mackiewicz P, Nadachowski A. 2020. The impact of major warming at 14.7 ka on environmental changes and activity of Final Palaeolithic hunters at a local scale (Orawa-Nowy Targ Basin, Western Carpathians, Poland) *Archaeological and Anthropological*

*Sciences* 12(3): 66.

Nadachowski A, Valde-Nowak P. 2015. New Late Pleistocene faunal assemblages from Podhale Basin, Western Carpathians, Poland: preliminary results *Acta Zoologica Cracoviensia* 58: 181–194.

## Trinkaus E, Haduch E, Valde-Nowak P, Wojtal P. 2014. The Obłazowa 1 pollical distal phalanx and Late Pleistocene distal thumb proportions, *HOMO – Journal of Comparative Human Biology* 65(1): 1–12.

Valde-Nowak P, Kraszewska A, Cieśla M, Nadachowski A. 2018. Late Magdalenian campsite in a rock shelter at the Obłazowa Rock. In: Valde-Nowak P, Sobczyk K, Nowak M, Żrałka J, eds. Multas per Gentes et Multa per Saecula. Anici magistro et college suo ioanni Christopho Kozłowski dedicant, Institute of Archeology, Jagiellonian University, Kraków, 175-183.

Valde-Nowak P, Nadachowski A. 2014. Micoquian assemblage and environmental conditions for the Neanderthals in Obłazowa Cave, Western Carpathians, Poland, *Quaternary International* 326-327:146 –156.

Valde-Nowak P, Nadachowski A, Madeyska T. (eds). 2003. Obłazowa Cave: human activity, stratigraphy and palaeoenvironment. Institute of Archaeology and Ethnology Polish Academy of Sciences.

Valde-Nowak P, Nadachowski A, Wolsan M. 1987. Upper Palaeolithic boomerang made of a mammoth tusk in south Poland, *Nature* 329: 436–438.

## Obłazowa 2, Western Carpathians, Nowa Biała, Poland

Obłazowa 2 site (49°25’48”N, 20°09’36”E) is located in Obłazowa Rock, only 50 m from the main entrance to the Obłazowa Cave. It is an artificial shelter, about 7 m long and 3 m wide, with a big opening facing east. Probably in 1920s and 1930s the eastern part of Obłazowa Rock was exploited as a quarry and at that time an artificial cave was made. The shelter was established in a natural fissure (with an original width of approx. 0.5 m) developed at the contact zone between the crinoid limestone and red nodular limestone and filled with large amount of sharp-edged limestone rubble and loamy clay, intense brownish-red in color. The fossil-bearing sediment contained regularly distributed sub-fossil bones and teeth. The rich fauna of vertebrates consists of very rare fishes, amphibians and reptiles and very abundant remains of birds (38 taxa) and mammals (33 species) (total NISP = 9490; MNI = 4636) (Nadachowski *et al.*, 1993). The faunal composition indicates a park-steppe and/or forest-steppe biomes, abundant in humid environments. The fauna is dated to the middle part of MIS 3 (the date was obtained from a fox mandible: OxA-3696, 33,430 ± 1230 BP).

**References**

Nadachowski A, Harrison DL, Szyndlar Z, Tomek T, Wolsan M. 1993. Late Pleistocene vertebrate fauna from Obłazowa 2 (Carpathians, Poland): paleoecological reconstruction, *Acta Zoologica Cracoviensia* 36(2): 281–290.
